# Draft Genome Sequences of Two Strains of Serratia spp. from the Midgut of the Malaria Mosquito Anopheles gambiae

**DOI:** 10.1128/genomeA.00090-15

**Published:** 2015-03-12

**Authors:** Dong Pei, Casey Hill-Clemons, Guillaume Carissimo, Wanqin Yu, Kenneth D. Vernick, Jiannong Xu

**Affiliations:** a Biology Department, Molecular Biology Program, New Mexico State University, Las Cruces, New Mexico, USA; b Pasteur Institute, Paris, France; c Department of Microbiology, University of Minnesota, Minneapolis, Minnesota, USA

## Abstract

Here, we report the annotated draft genome sequences of two strains of Serratia spp., Ag1 and Ag2, isolated from the midgut of two different strains of Anopheles gambiae. The genomes of these two strains are almost identical.

## GENOME ANNOUNCEMENT

The genus Serratia consists of Gram-negative rod-shaped facultative anaerobes. Members of this genus share a variety of habitats, such as freshwater, soil, plants, and the gut of invertebrates and vertebrates. The genus includes many human pathogenic strains (1). In addition, other strains have been investigated, for example, a strain from the gut of water flea Daphnia magna (2), and a strain that is associated with leaf-cutter ant fungus gardens (3). The mosquito gut ecosystem accommodates a dynamic microbiome (4, 5). Serratia odorifera could significantly enhance susceptibility to dengue virus DENV-2 in Aedes aegypti (6). In anopheline mosquitoes, Serratia marcescens has been found to be able to inhibit Plasmodium development in Anopheles gambiae (7, 8).

The Serratia sp. strain Ag1 was isolated from the Ngousso strain of A. gambiae that was originally collected in Cameroon in 2006, and maintained in the Vernick lab, the Institut Pasteur, Paris, France. The Serratia sp. strain Ag2 was isolated from the A. gambiae G3 strain in the Xu lab at New Mexico State University, Las Cruces, NM, USA. The G3 strain was derived from a collection originally in MacCarthy Island, the Gambia, and colonized in 1975. The genome was determined by Illumina paired-end technology at Genewiz, Inc. A total of 6.1 M reads for strain Ag1 and 4.5 M reads for strain Ag2 were generated. *De novo* assembly was performed using CLC genomic workbench (v7.0.4). The assembly of strain Ag1 contains 110 contigs totaling 5.35 Mbp. The assembly of strain Ag2 contains 109 contigs totaling 5.32 Mbp. The genomes were annotated by the NCBI Prokaryotic Genome Automatic Annotation Pipeline. The Ag1 genome includes 4,773 coding sequences (CDS) and 85 RNA genes. The Ag2 genome includes 4,706 CDS and 85 RNA genes. Genome comparison indicated the common origins of the isolates. Approximately 98.2% of the Ag1 genome has identical counterparts in Ag2, whereas 98.7% of the Ag2 genome has identical counterparts in Ag1. The differences between the two strains were minor. For example, contig 54 (9,245 bp) in Ag1 has no counterpart in Ag2, and contig 12 (18,403 bp) and contig 78 (15,537 bp) from Ag2 are not present in Ag1. Intriguingly, the two almost identical strains are associated with different mosquito colonies originally from different parts of Africa, and the two colonies were never maintained in the same insectary. This strongly suggests that the bacteria are persistently associated with the natural mosquito microbial flora in nature. As a member of the gut flora of Ngousso mosquitoes, strain Ag1 may be involved in the susceptibility to Plasmodium and O’nyong-nyong virus (9).

Recently, several genomes of mosquito associated bacteria have been sequenced, including Elizabethkingia anophelis (10, 11), Asaia spp. (12), Enterobacter spp. (13), Pseudomonas spp. (14), and Spiroplasma diminutum and S. taiwanense (15, 16). The available genomes will facilitate to characterize the structure and function of the mosquito microbiome.

### Nucleotide sequence accession numbers.

The genome sequences have been deposited at GenBank under the accession numbers JQEI00000000 (Serratia sp. Ag1) and JQEJ00000000 (Serratia sp. Ag2).
